# Beyond Burning Mouth Pattern: A Veiled Masquerading Oral Health Anxiety Concern

**DOI:** 10.7759/cureus.89712

**Published:** 2025-08-09

**Authors:** Srinivas Kandula, Atul Bajoria, Sangamesh NC, Silpiranjan Mishra, Tania Biswas

**Affiliations:** 1 Department of Oral Medicine, Manipal Hospital, Bangalore, IND; 2 Department of Oral Medicine and Radiology, Kalinga Institute of Dental Sciences, Bhubaneswar, IND

**Keywords:** burning mouth syndrome, cognitive behavioural therapy., cyberchondria, hypochondriasis, illness anxiety disorder, oral health anxiety, oral medicine, psychogenic pain, somatic symptom disorder

## Abstract

Burning mouth syndrome (BMS) is a chronic oral pain condition characterized by a burning sensation in the absence of clinical lesions. This report discusses two cases where oral burning and anatomical variations led to severe oral health anxiety, cyberchondria, and psychiatric symptoms. Despite no clinical pathology, both patients developed carcinophobia due to misinterpretation and misinformation. Multidisciplinary management involving oral medicine, psychiatry, and cognitive behavioral therapy (CBT) proved effective. The cases underscore the need for clinician awareness and psychological insight in managing psychogenic oral complaints.

## Introduction

Health anxiety, previously termed hypochondriasis, is characterized by excessive fears or worries about having a serious illness despite medical reassurance. In the Diagnostic and Statistical Manual of Mental Disorders, fifth edition (DSM-5), hypochondriasis has been replaced by somatic symptom disorder (SSD) and illness anxiety disorder (IAD), with high health anxiety as a key criterion. These conditions are often associated with significant distress, frequent medical consultations, and functional impairment [1].

In the realm of oral health, this anxiety can become specifically fixated on the fear of developing oral cancer, a phenomenon referred to as oral cancer phobia. Fear of oral cancer can cause patients to either avoid screenings altogether or frequently visit multiple doctors unnecessarily. This anxiety can also negatively impact treatment outcomes, such as reduced responsiveness to therapy. Recognizing and understanding these behavioral patterns is essential for developing better prevention and management strategies, including incorporating psychological support and behavioral therapy [2].

Body dysmorphic disorder (BDD) is a psychiatric condition characterized by an obsessive preoccupation with perceived defects or flaws in physical appearance, which are either minor or not observable to others. This excessive concern often leads to repetitive behaviors such as mirror checking, seeking reassurance, and frequent comparisons with others, significantly impairing social, occupational, and personal functioning. Although traditionally associated with dermatology and cosmetic concerns, BDD can also manifest in dental and oral health settings, where patients fixate on normal anatomical variations in the mouth. This underscores the need for dental professionals to recognize the psychological underpinnings of such complaints to ensure appropriate interdisciplinary management and prevent unnecessary interventions [3].

Hypochondriasis, now largely reclassified under illness anxiety disorder (IAD) and somatic symptom disorder (SSD) in the DSM-5, is characterized by an excessive and persistent fear of having a serious illness despite medical reassurance and absence of significant pathology. Individuals with this condition misinterpret normal bodily sensations as signs of severe disease, leading to heightened anxiety, frequent medical consultations, and substantial functional impairment. The article emphasizes that while DSM-5 separates IAD and SSD based on symptom intensity and health preoccupation, both disorders represent a continuum of maladaptive health anxiety, requiring careful clinical differentiation and a shift from purely medical to biopsychosocial management strategies [4].

Cognitive behavioral therapy (CBT) is a psychological intervention that has shown effectiveness across a range of psychiatric and physical health conditions, including depression, anxiety, and chronic pain disorders. In recent years, its application has extended into dental settings, addressing psychosomatic complaints such as temporomandibular disorders (TMD), dental anxiety, burning mouth syndrome (BMS), and other medically unexplained oral symptoms. The core principle of CBT involves modifying maladaptive thoughts and behaviors to alleviate emotional and physical distress. Within dental practice, CBT has been delivered using various techniques, like relaxation training, biofeedback, cognitive restructuring, and exposure therapy, to enhance patient outcomes, especially for those who present with psychosomatic symptoms but lack identifiable clinical findings. Given the growing relevance of psychological factors in dental health, training dental professionals in CBT methods is becoming increasingly important to bridge the gap between psychological and oral health care [5].

The aim of this article is to explore the psychosomatic dimensions of BMS and its association with health anxiety, cyberchondria, and psychiatric manifestations in patients without detectable clinical pathology. To assess the severity of the oral burning sensation, the visual analog scale (VAS) was employed as a subjective pain measurement tool. Patients were provided with a 10-centimeter horizontal line, with the left end marked as “0 - No burning sensation” and the right end marked as “10 - Worst imaginable burning sensation.” Each patient was asked to indicate on the line the point that best represented the intensity of their burning sensation at the time of evaluation. This study also aims to highlight the role of oral medicine specialists in identifying, managing, and providing multidisciplinary care for patients presenting with psychogenic oral complaints and to evaluate the effectiveness of integrating cognitive behavioral therapy (CBT) and psychiatric support in alleviating symptoms and improving quality of life for patients with BMS-like presentations. 

## Case presentation

Case 1

A 48-year-old male, employed as a government clerk, presented with a nine-month history of persistent burning sensation in the anterior two-thirds of the tongue and inner aspects of the lips. The patient described the burning as a 6-7/10 on the pain scale, more intense in the evening, and continuous in nature. He denied aggravation with spicy food, brushing, or thermal stimuli. There were no associated symptoms such as ulceration, dryness, or difficulty in eating. He had no habits such as smoking or tobacco use and maintained good oral hygiene.

The patient had consulted multiple healthcare providers, including dentists, ear, nose, throat (ENT) specialists, and general physicians. Treatments included antifungal mouth rinses, vitamin B-complex supplements, and clonazepam, but none provided lasting relief. Basic blood reports and imaging had been unremarkable. Despite repeated reassurances about the benign nature of his symptoms, his frustration and health anxiety grew.

In his quest for answers, the patient began extensively searching the internet, encountering alarming information linking oral burning to oral cancer. He became convinced that his symptoms were indicative of malignancy and began examining his mouth multiple times a day with a flashlight and mirror. This led to increasing anxiety, sleep disturbances, and avoidance of social interactions.

**Figure 1:**
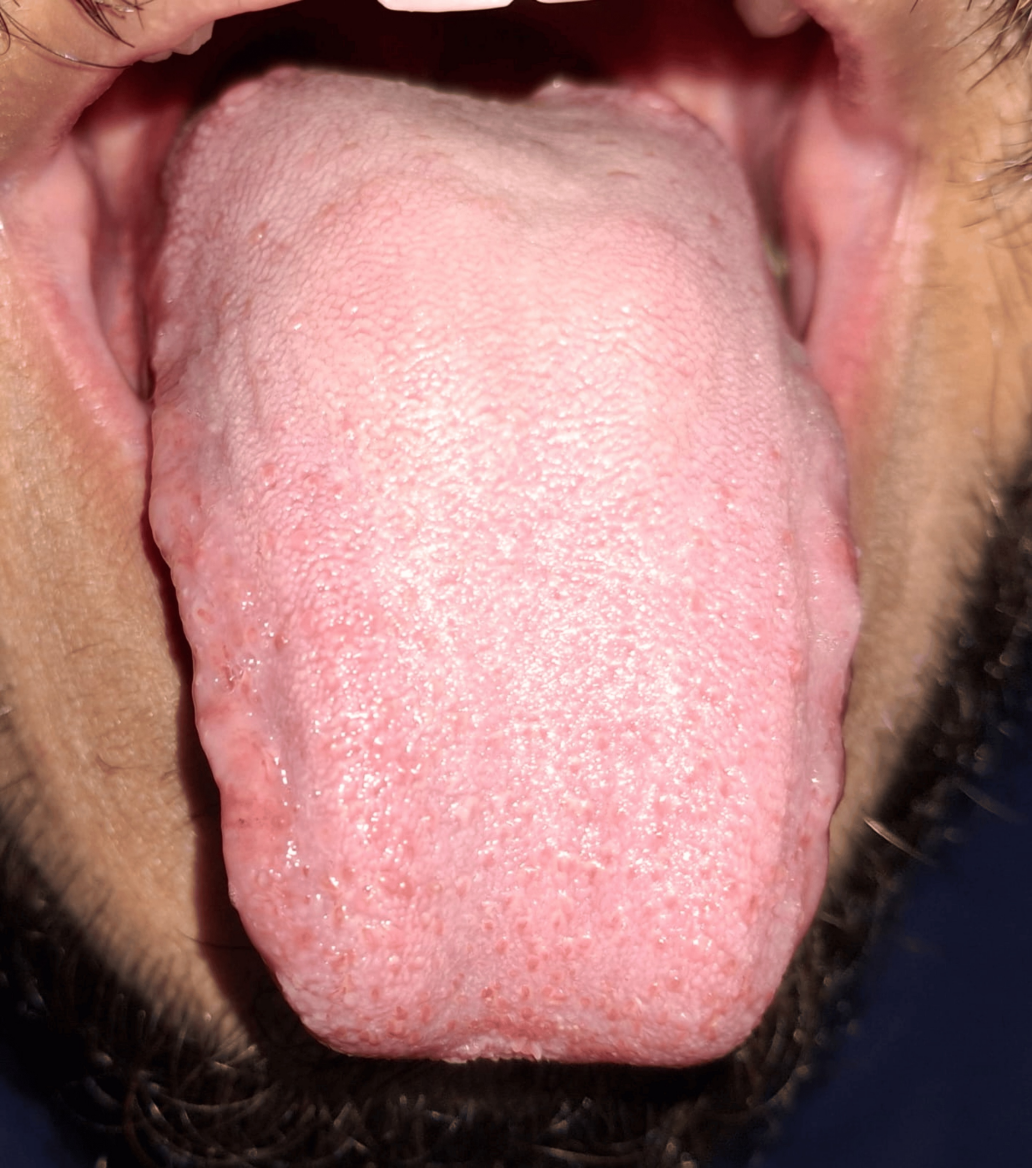
Normal tongue; no visible lesions

**Figure 2:**
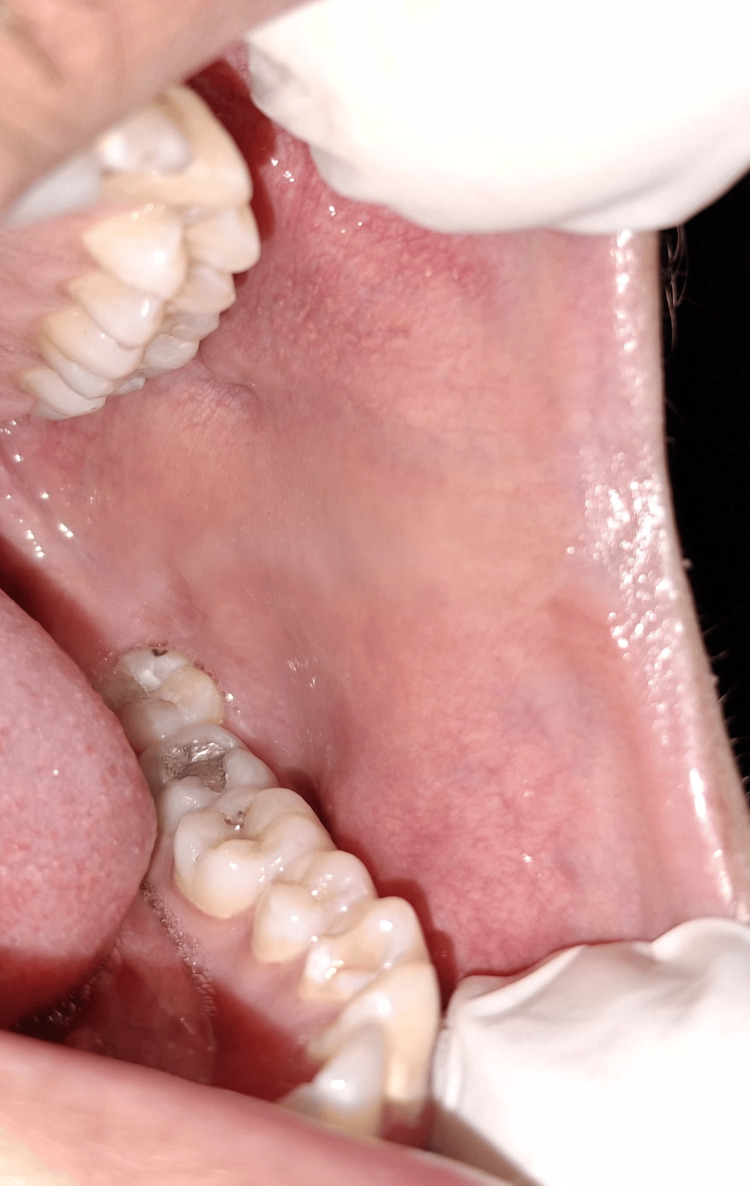
Normal left buccal mucosa

**Figure 3:**
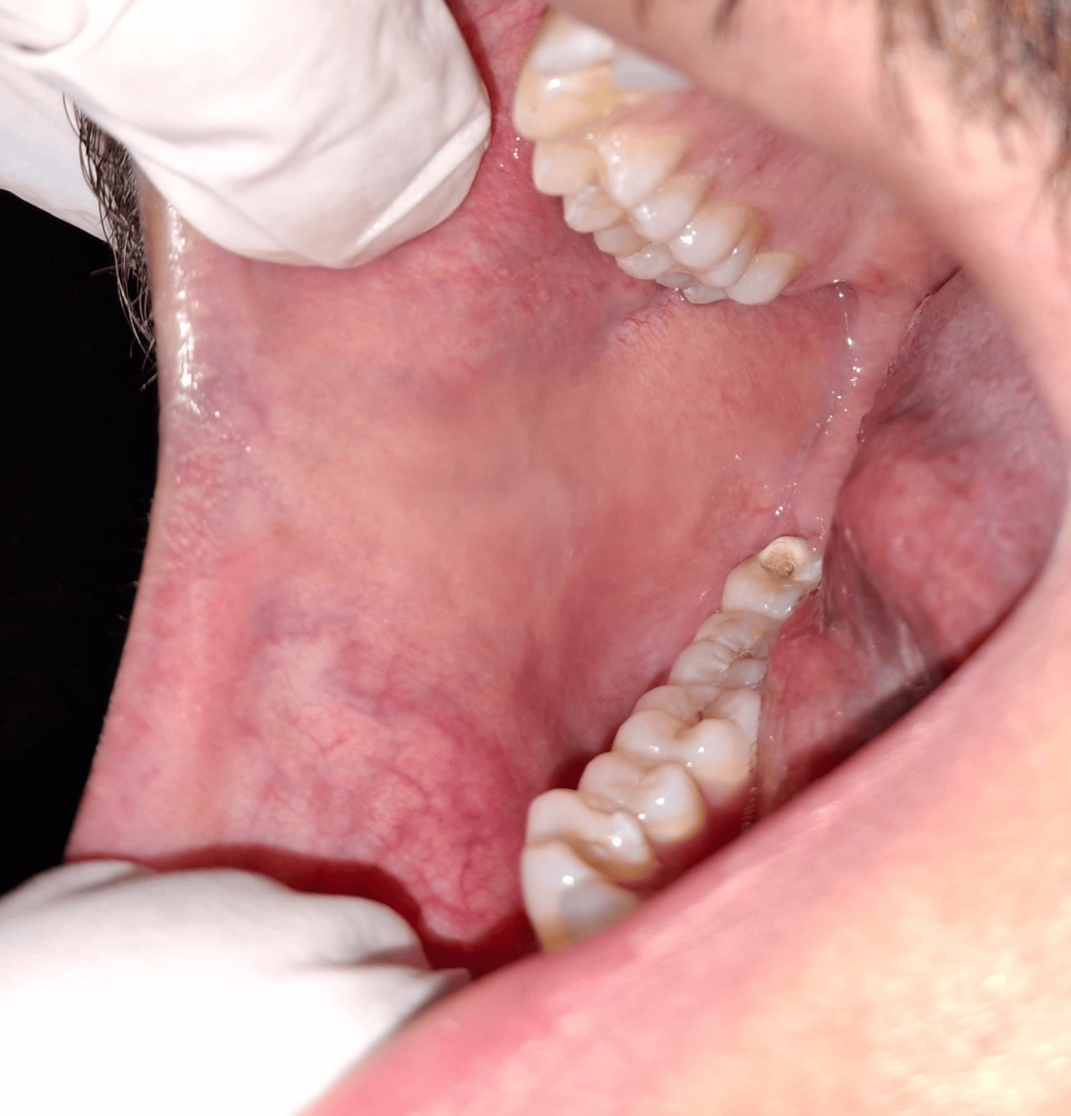
Normal right buccal mucosa

Intraoral examination revealed no mucosal lesions, ulcerations, or signs of infection. The tongue appeared normal with no signs of erythema or atrophy. No lymphadenopathy was noted. Salivary flow was within normal limits. No signs of geographic tongue or candidiasis were observed (Figures 1-3).

Due to the escalating anxiety and obsessive mouth-checking behavior, a psychiatric referral was made. The psychiatrist diagnosed him with health anxiety disorder (hypochondriasis) with obsessive-compulsive features. He also exhibited signs of mild depression and reported significant emotional distress, including hopelessness and irritability.

For management, the patient was provided with a detailed explanation regarding the diagnosis of burning mouth syndrome (BMS), emphasizing its benign and non-cancerous nature to alleviate his anxiety. Educational materials were shared to help him understand the condition better and to discourage excessive internet searches and self-diagnosis, which had been contributing to his health anxiety. Psychiatric management was initiated with a low dose of amitriptyline (10 mg at bedtime) along with clonazepam 0.25 mg once daily to address his associated anxiety and sleep disturbances. Additionally, weekly cognitive behavioral therapy (CBT) sessions of 30 minutes were scheduled to target his obsessive health-related behavior and underlying anxiety. The patient was also advised to attend regular dental follow-ups for continuous reassurance and monitoring, and to build trust in professional care over self-research.

Within four weeks, the patient reported slight improvement in his burning sensation (VAS score reduced to 2/10) and improved sleep. By the end of three months, his anxiety levels had significantly reduced, he no longer insisted on cancer screening, and his symptoms were manageable. He resumed work and social activities with greater confidence.

Case 2

A 35-year-old female presented with a six-month history of mild, intermittent burning sensations localized to the lateral borders of the tongue. The discomfort was described as a tingling or stinging sensation occurring mostly in the evenings, with a VAS score of 4/10, and it was occasionally accompanied by a dry mouth. She reported the onset of symptoms after noticing a dark pigmentation on her tongue while using a hand mirror. She had no history of smoking, alcohol, or pan chewing. The patient denied any change in taste perception or tongue movement.

She admitted to repeatedly examining her mouth in various lighting conditions and under magnification. Her concern centered on the shape and color of the tongue papillae, which she believed had "changed." She reported Googling her symptoms extensively and feared she might have early-stage cancer, despite being asymptomatic otherwise. The obsession led to disturbed sleep and frequent medical consultations.

**Figure 4:**
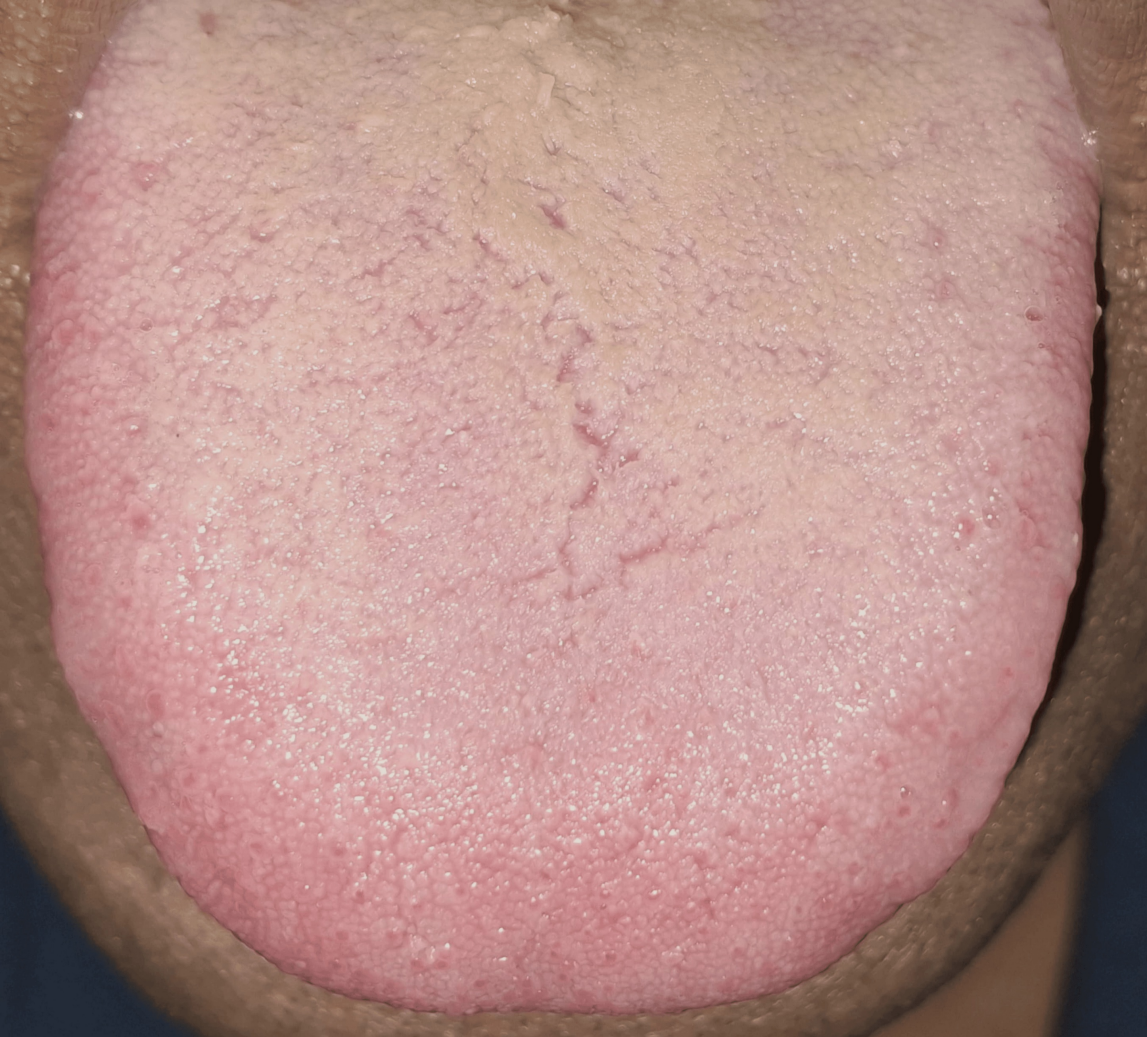
Fissured tongue

**Figure 5:**
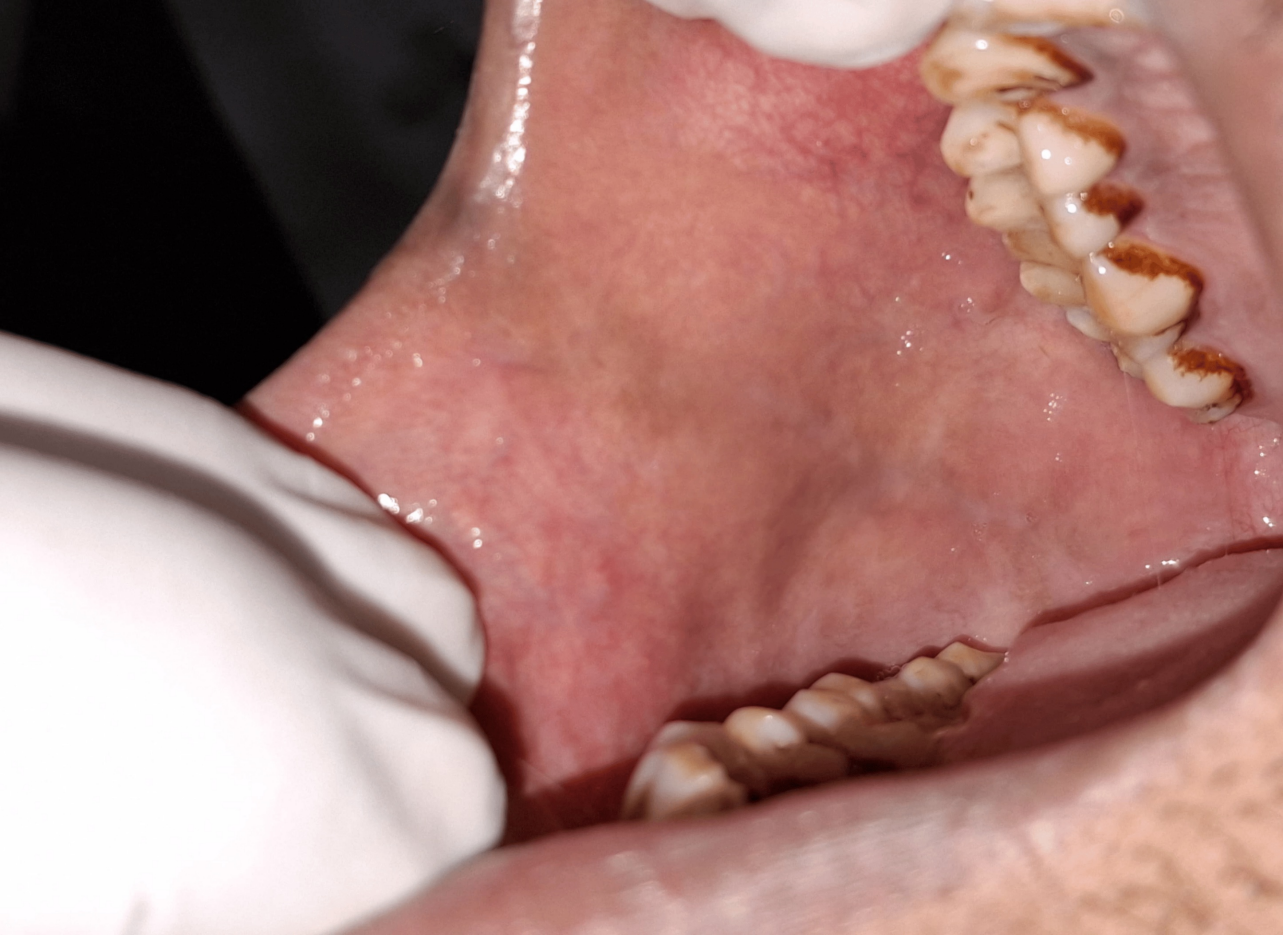
Physiological hyperpigmentation of right buccal mucosa

Intraoral examination revealed the presence of deep fissures across the dorsal surface of the tongue, indicative of a fissured tongue, along with melanin pigmentation along the attached gingiva. There were no ulcers, white patches, or any clinical signs suggesting malignancy. However, the examination proved to be challenging due to the patient’s persistent belief that these physiological features were pathological. Despite thorough counseling and reassurance regarding the benign nature of the findings, she repeatedly expressed concern and questioned whether the condition could be cancerous (Figures 4, 5).

Laboratory investigations were conducted to rule out any underlying nutritional deficiencies that could contribute to the patient's symptoms. A complete blood count (CBC) was performed and found to be within normal limits. The peripheral smear revealed normocytic, normochromic red blood cells, indicating no significant hematological abnormalities (Table 2). Levels of vitamin B12, serum iron, ferritin, and folic acid were all within normal range (Tables 1, 3), effectively excluding common nutritional causes for the patient's concerns.

**Table 1:** The vitamin B12 assay of the patient was within the normal range

INVESTIGATION	RESULT
Vitamin B12(CYANOCONALAMIN)	452.00 (pg/ml)

**Table 2:** A complete blood count shows normal hematological parameters MCV: Mean corpuscular volume, MCH: Mean corpuscular hemoglobin, MCHC: Mean corpuscular hemoglobin concentration

PARAMETER	RESULT
Haemoglobin	12.90 (gm/dl)
Total leukocyte count	7.50 (10^3/µL)
Neutrophils	56.50 (%)
Lymphocytes	36.10 (%)
Eosinophils	3.10 (%)
Monocytes	4.90 (%)
Basophils	0.40 (%)
Absolute Neutrophils	2.89 (10^3/µL)
Absolute lymphocytes	3.31 (10^3/µL)
Absolute Eosinophils	0.37 (10^3/µL)
Absolute Monocytes	0.86 (10^3/µL)
Absolute Basophils	0.07 (10^3/µL)
Total Red Blood Count	4.68 (million/cu mm)
Hematocrit	45.90 (%)
MCV	78.80 (fl)
MCH	27.70 (pg)
Platelet	274 (10^3/µL)
MCHC	34 g/dl

**Table 3:** Iron study panel showing values within normal range

SERUM IRON PROFILE	RESULT
Iron	102.00 (µg/dl)
Total iron binding capacity	324.00 (µg/dl)
Transferrin saturation	38.33 (%)
Ferritin	222.00 (ng/dl)

As there were no hematological abnormalities observed, we included a differential diagnosis of atypical odontalgia and anemic stomatitis.

The management of this case was approached with a multidisciplinary strategy, emphasizing both patient reassurance and psychological intervention. During the consultation, visual aids and diagrams were employed to help the patient understand that the fissured tongue and melanin pigmentation were benign, normal anatomical variants. The patient was advised to reduce the frequency of oral self-examinations and was counseled about the psychological implications of obsessive health-related behaviors. Recognizing the underlying anxiety and health fixation, she was referred to a mental health professional, where a diagnosis of generalized anxiety disorder (GAD) was confirmed. Psychological counseling was initiated, with a focus on cognitive restructuring to address her intrusive thoughts and misinterpretation of normal oral features as pathological. She was also advised to limit internet searches related to medical conditions (cyberchondria management). In parallel, clinicians involved in her care were educated on how to effectively communicate benign findings and avoid unnecessary investigations that could reinforce the patient’s fears.

Over the course of the eight-week follow-up, the patient reported a gradual reduction in her self-checking behavior and a noticeable improvement in her sleep and mood. Her anxiety regarding oral cancer significantly diminished, and she no longer sought repeated consultations. By the end of three months, she expressed greater confidence in her health, reduced reliance on internet-based self-diagnosis, and was able to resume her normal routine without distress. The score improved to 1/10, correlating with a significant decline in burning episodes and improved overall comfort. This outcome underscores the critical role of empathetic communication, education, and timely psychiatric referral in managing psychogenic oral pain presentations.

## Discussion

The updated Cochrane review by Zakrzewska et al. reinforces the therapeutic challenges associated with Burning Mouth Syndrome, highlighting that most pharmacologic interventions yield inconsistent or short-term results. Notably, only topical clonazepam and a single psychological therapy trial demonstrated lasting benefit [6]. This aligns with our findings, emphasizing that psychological factors often underpin BMS symptoms and that incorporating cognitive and behavioral strategies, rather than relying solely on medications, may offer more sustainable outcomes in such patients.

Burning mouth syndrome (BMS) presents a complex clinical challenge, often characterized by chronic oral burning sensations without accompanying clinical or laboratory findings. Grushka et al. emphasized that BMS is more prevalent among postmenopausal women and frequently coexists with symptoms such as dry mouth and altered taste. While psychological dysfunction, such as anxiety and depression, has been proposed as a contributing factor, it is now understood that such psychological manifestations may also be consequences of chronic pain rather than its cause. This underscores the bidirectional relationship between chronic orofacial discomfort and mental health and also suggests the involvement of cranial nerve dysfunction, especially those associated with taste. Pharmacologic management using low-dose benzodiazepines, tricyclic antidepressants, or anticonvulsants like gabapentin, along with topical capsaicin, has been found to alleviate symptoms in some patients [7].

In a pivotal study by Takenoshita et al. (2010), the psychiatric profiles of patients suffering from burning mouth syndrome (BMS) and atypical odontalgia (AO) referred from psychiatric to dental facilities were evaluated, highlighting significant differences in diagnostic classifications. BMS patients were more frequently diagnosed with mood or affective disorders (F3 classification), whereas AO patients showed a higher prevalence of neurotic, stress-related, and somatoform disorders (F4 classification). Interestingly, over 50% of BMS patients and one-third of AO patients had no specific psychiatric diagnoses, emphasizing the diagnostic ambiguity surrounding these chronic pain conditions. This underscores the complex interplay of psychological and somatic elements in BMS and AO and the need for a multidisciplinary approach in their assessment and management, which aligns with the challenges observed in the current study cohort [8].

As demonstrated by Naidu et al. (2008), many patients, particularly those anxious and desperate, may turn to unqualified practitioners (“quacks”), receiving inappropriate treatments that fail to address their underlying concerns, often exacerbating their health fears and delaying proper diagnosis [9].

Tanis et al. (2016) found that patients with elevated health anxiety not only search for health information online more frequently but also report significantly lower satisfaction with their medical consultations. Intriguingly, the more extensively these individuals researched before the appointment, the less satisfied they felt about consultation length, especially if they were already health anxious. This suggests that the internet can exacerbate dissatisfaction among anxious patients, reinforcing self-diagnosis and mistrust [10]. In our study, similar patterns emerged: individuals with oral health anxiety engaged in excessive internet searching, leading to increased dissatisfaction with specialists and frequent doctor-shopping. These findings underscore the critical need for clinicians, particularly oral medicine specialists, to proactively address misinformation, dedicate adequate time for patient dialogue, and preempt the reassurance trap by offering structured follow-up and clear guidance.

McMullan et al. (2019) conducted a comprehensive meta-analysis examining the link between health anxiety and online health-related behavior. Their findings revealed a strong positive relationship: individuals with higher health anxiety were significantly more likely to search the internet for medical symptoms and display cyberchondria, a pattern of escalating anxiety through online information. Notably, the study found that emotion dysregulation and negative cognitive biases mediate this relationship, leading to worsening distress despite increasing symptom-checking behavior [11]. Within our study, patients with oral health complaints mirrored these behaviors, engaging in repetitive self-examination and reliance on unvetted web sources, often resulting in elevated anxiety, mistrust of clinician reassurance, and unnecessary consultations. This evidence reinforces the importance of timely psychological assessment, patient education on information evaluation, and structured clinical follow-up to mitigate the harmful cycle of anxiety-seeking.

Błachnio et al. (2022) shed light on the emotional mechanisms underpinning the relationship between health anxiety and cyberchondria. They found that individuals with poor emotional regulation and a tendency toward negative cognitive bias are more susceptible to escalating anxiety through repetitive online symptom searching, despite a lack of clinical validation. These emotional vulnerabilities act as mediators, converting benign sensations into distressing health fears [12]. In our case, patients fixated on normal oral variations and engaged in obsessive self-monitoring via mirrors or flashlights, often compounded by internet misinformation. This supports the notion that emotional dysregulation, not just information exposure, intensifies health anxiety. Consequently, therapeutic strategies should extend beyond patient education to include emotional coping skills training and targeted behavioral interventions.

White and Horvitz (2009) introduced the term “cyberchondria” to describe the phenomenon where web searches for medical symptoms escalate users’ concerns about their health. Their research demonstrated how innocuous queries can lead to increased anxiety through exposure to rare and severe conditions early in search results. This behavior pattern was clearly observed in our patients, who began with benign oral symptoms but, after repeated online searches, developed intense fears of malignancy, including oral cancer. This escalation loop not only heightened their psychological distress but also reduced the effectiveness of clinical reassurance. These findings emphasize the need for clinicians to actively guide anxious patients away from unfiltered health content and toward credible resources, while also addressing their underlying anxiety through counselling or cognitive-behavioral strategies [13].

The longitudinal study by Fink et al. (2010) demonstrated that patients with health anxiety incur significantly higher health care costs and report poorer self-rated health over time compared to non-anxious individuals. Despite frequent medical consultations and investigations, these patients often fail to achieve reassurance, leading to persistent distress and increased reliance on the healthcare system. The study emphasized the importance of early identification and targeted psychological interventions to mitigate the progression and economic burden of health anxiety [4]. These observations parallel the findings of our study, where patients with persistent oral symptoms, often without clinical pathology, displayed repeated care-seeking behavior, self-diagnosis via online sources, and escalating anxiety. This highlights the pressing need to integrate psychosomatic evaluation and counselling within dental practice, particularly for patients exhibiting health-anxious behaviors in the context of oral sensations or benign anatomical variations.

Morales‑Gómez et al. (2025) conducted a comparative evaluation of telediagnosis accuracy between oral medicine specialists and general dental practitioners using images of mucosal lesions. The results demonstrated a striking discrepancy: specialists achieved an 86.5% diagnostic accuracy rate, compared with only 49.2% among generalists. This significant performance gap underscores specialists’ superior training in distinguishing benign oral variants and avoiding unnecessary biopsies or referrals based on visual assessment alone. In the context of our findings, this emphasizes the pivotal role of oral medicine specialists, especially in settings where face-to-face consultations may be limited. Their ability to accurately interpret normal anatomical variations, educate patients about benign findings, and prevent overtreatment reinforces the need for early specialist referral in cases of oral health anxiety and unexplained symptoms [14].

In their 2023 article, Poornachitra and Narayan proposed a practical algorithm for managing dental patients with underlying mental health conditions within special care settings. This framework emphasizes early screening, mental health triage, and tailored oral care plans, recommending coordination of CBT, pharmacologic therapy, and referral to behavioral health professionals when needed. The authors underscore the importance of empathetic communication and structured follow-up to mitigate anxiety-driven behaviors such as doctor-shopping or self-diagnosis [15]. These principles resonate with our findings: patients in our study often presented with anxiety-laden oral complaints, fueled by misinformation and poor emotional coping. Their model supports the integration of mental health evaluation into routine dental protocols and suggests that similar structured approaches can enhance care for patients with psychogenic oral concerns.

Lotto et al. (2023) conducted a content analysis of online oral health misinformation and found that social media platforms often propagate exaggerated or false claims, especially regarding oral cancer risk and unproven treatment modalities. A significant proportion of content lacked credible dental or scientific backing, and many posts were authored by non-expert sources with financial or sensational motives. This environment fosters excessive health anxiety and misinformation-driven behavior, such as self-examination, unnecessary consultations, and fear of normal oral variations [16]. These findings align closely with observations in our study, where patients frequently turned to unverified online information, escalating fears about benign anatomical features. The study underscores the urgent need for dental professionals to actively debunk misinformation, offer accurate educational resources, and preemptively counsel anxious patients to prevent digital-driven health distress.

A comprehensive 2022 systematic review by Tan et al., published in Cephalalgia, provides a robust evaluation of treatments for burning mouth syndrome (BMS). The analysis highlights that, while pharmacologic agents such as clonazepam, low-dose antidepressants, and alpha-lipoic acid offer modest relief, their efficacy is inconsistent and often short-lived. Importantly, psychological interventions, particularly structured cognitive behavioral therapy (CBT), demonstrated more substantial and sustained benefits in reducing symptom severity and improving quality of life [17]. This underscores the therapeutic value of a biopsychosocial model and aligns with the psychosomatic components observed in BMS patients within our cohort. Given the limited long-term success of medication alone, our findings further support integrating mental health strategies into oral medicine care pathways to manage chronic unexplained oral pain effectively.

Toyofuku (2016) provides an in-depth overview of psychosomatic conditions encountered in dentistry, emphasizing that many oral complaints, such as burning sensations, dysesthesia, and unexplained mucosal discomfort, often lack identifiable organic pathology and are intertwined with psychological distress. The author highlights the importance of adopting a biopsychosocial framework, which integrates mental health assessment and cognitive-behavioral interventions alongside clinical examination. Particularly relevant is the recommendation for dentists and oral medicine specialists to be trained in recognizing health anxiety, somatic symptom disorders, and body dysmorphic tendencies, and to coordinate with mental health professionals for comprehensive care. These principles resonate strongly with our study findings, reinforcing the critical role of interdisciplinary collaboration, targeted psychological support, and patient-centered education in managing psychogenic oral complaints and reducing unnecessary healthcare-seeking behaviors [18].

Oral medicine specialists, with their interdisciplinary expertise, play a pivotal role not only in diagnosis but also in patient education, reassurance, and coordinated psychological referral. A biopsychosocial model of care, centered on empathy, clarity, and continuity, remains the cornerstone for managing such complex presentations, ultimately restoring both the patient’s oral comfort and emotional well-being. Hence, dental professionals reinforced education about the benign nature of oral findings, thus preventing unnecessary investigations and interventions. This coordinated approach, integrating dental, psychiatric, and behavioral care, proved pivotal in breaking the cycle of anxiety-driven symptom escalation and restoring patient confidence and quality of life.

## Conclusions

These two cases collectively highlight the urgent need for a more holistic, psychologically informed approach to oral healthcare. The mere absence of a lesion does not equate to the absence of disease, especially when the disease is driven by psychological distress and distorted perception. Normal anatomical variants, when misunderstood, can become sources of profound fear. Uninformed clinicians and misleading online content further exacerbate this fear, pushing patients into a spiral of health anxiety, depression, and social dysfunction. Oral medicine specialists must lead the way in addressing this emerging pattern through empathetic education, structured reassurance, and collaboration with mental health services. Only by treating both the symptom and the psyche can true healing begin in such cases. By employing validated assessment tools such as the visual analog scale (VAS) for symptom intensity, alongside measures of health anxiety and quality of life, clinicians can more accurately document treatment outcomes and patient progress. Importantly, this holistic approach prevented further reinforcement of carcinophobia and cyberchondria, restored patients’ emotional stability, and significantly improved their daily functioning and quality of life.
